# Supplementary figures and images for: NSD2 is a requisite subunit of the AR/FOXA1 neo-enhanceosome in promoting prostate tumorigenesis
Source: bioRxiv. 2024 Mar 29:2024.02.22.581560. Preprint. [Version 3] doi: 10.1101/2024.02.22.581560 (PMC10925163; doi:10.1101/2024.02.22.581560)

# Figure S1

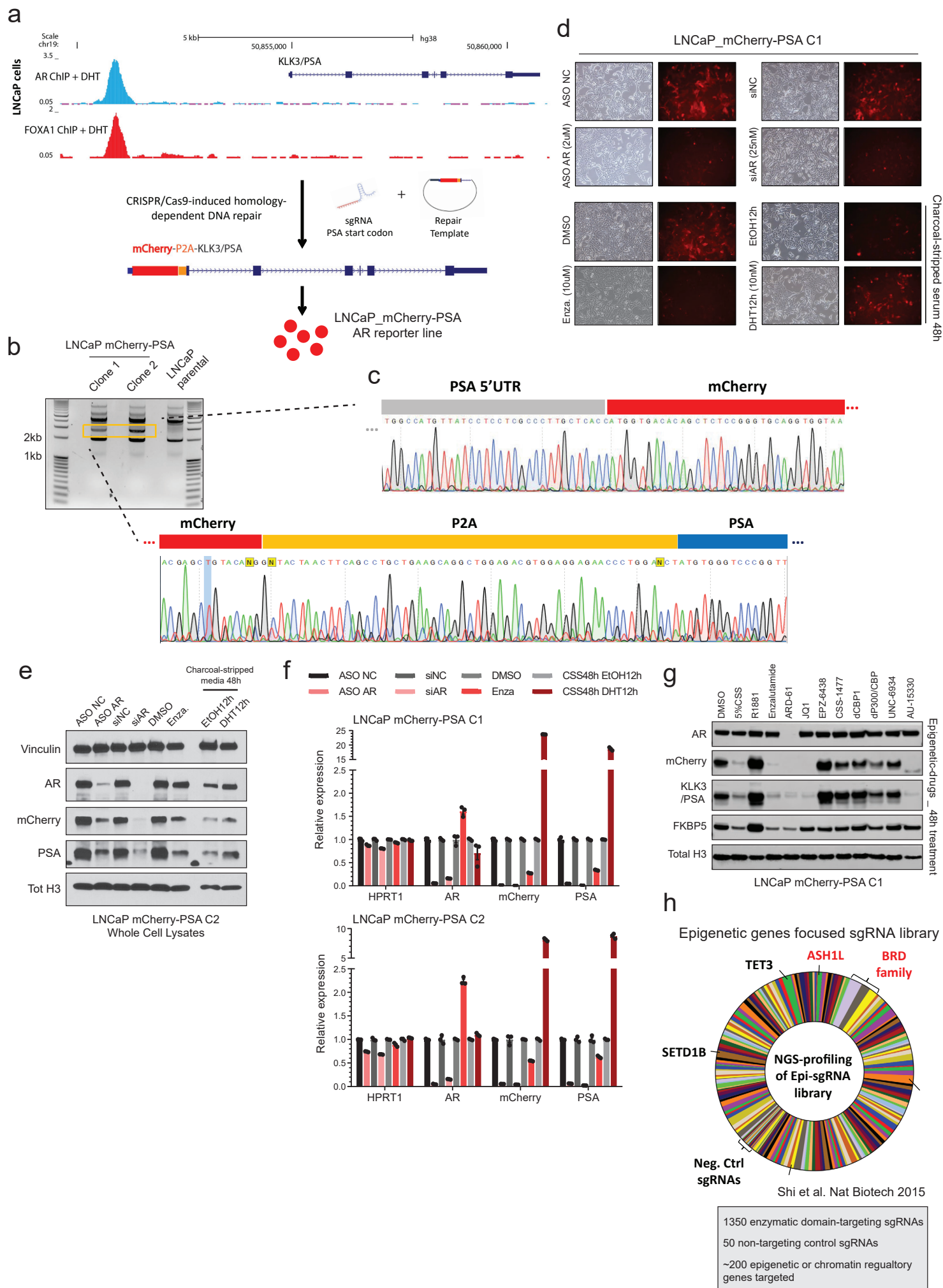

# Figure S2

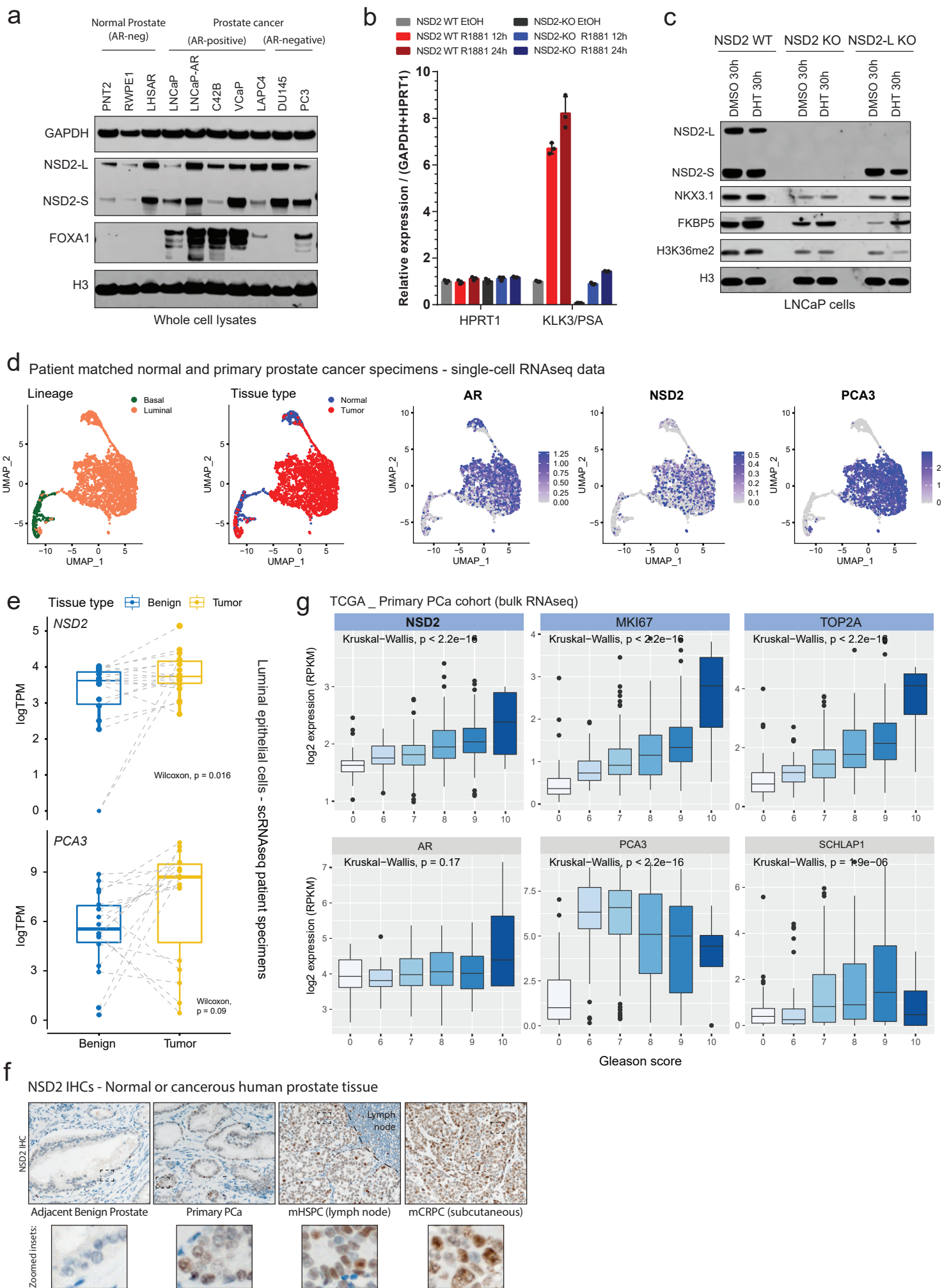

**Figure S3**

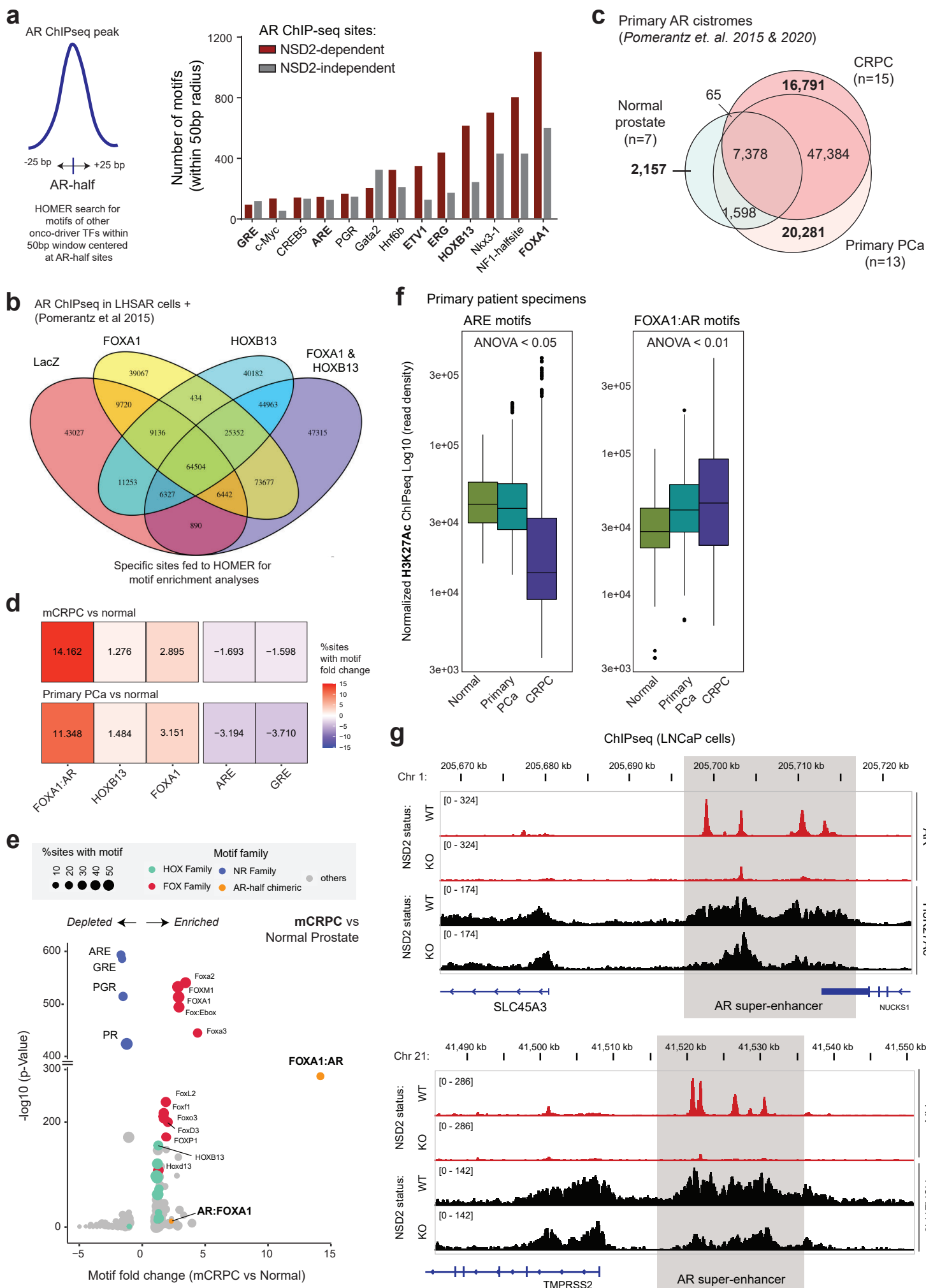

**Figure S4**

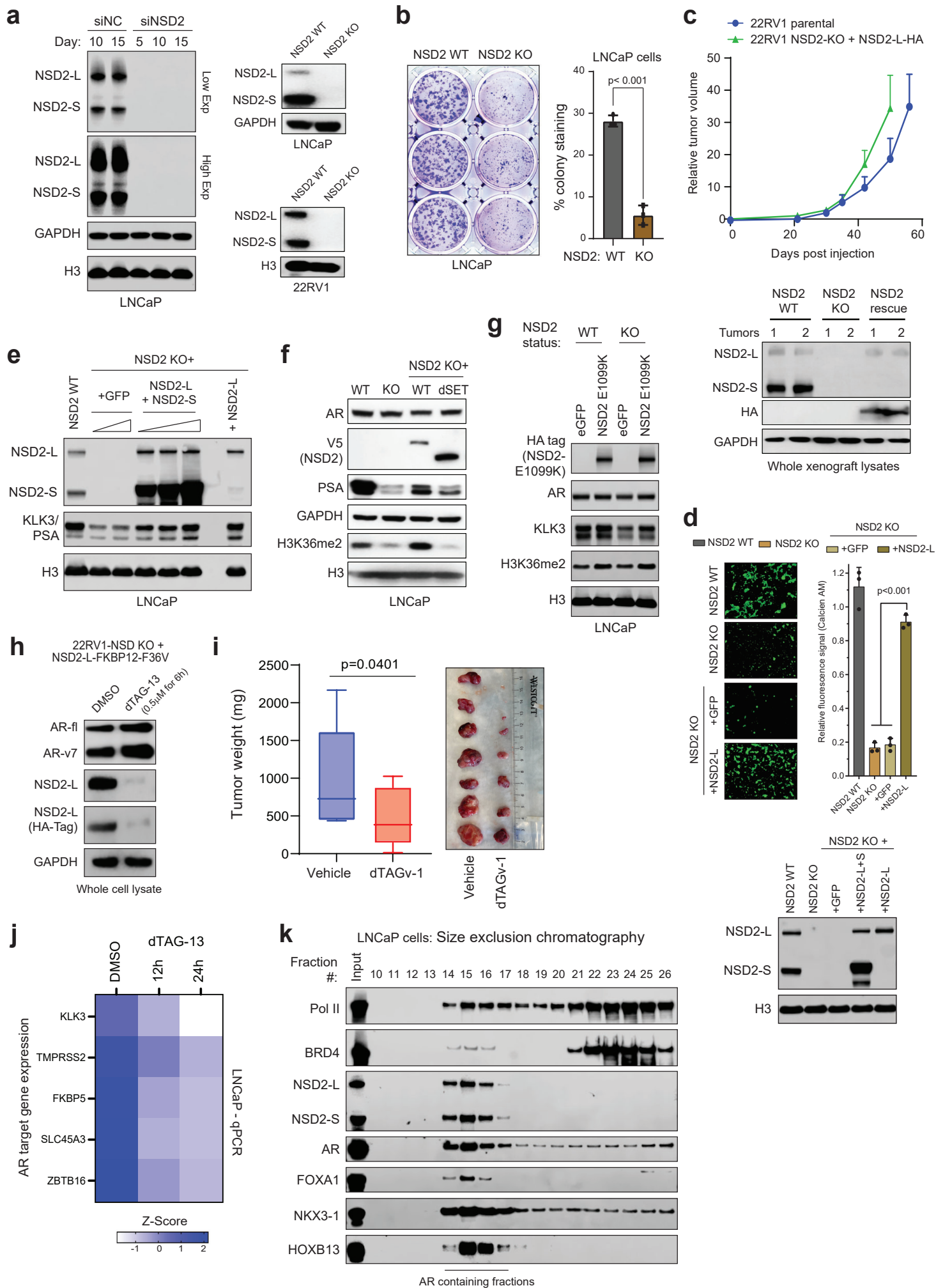

Figure S5

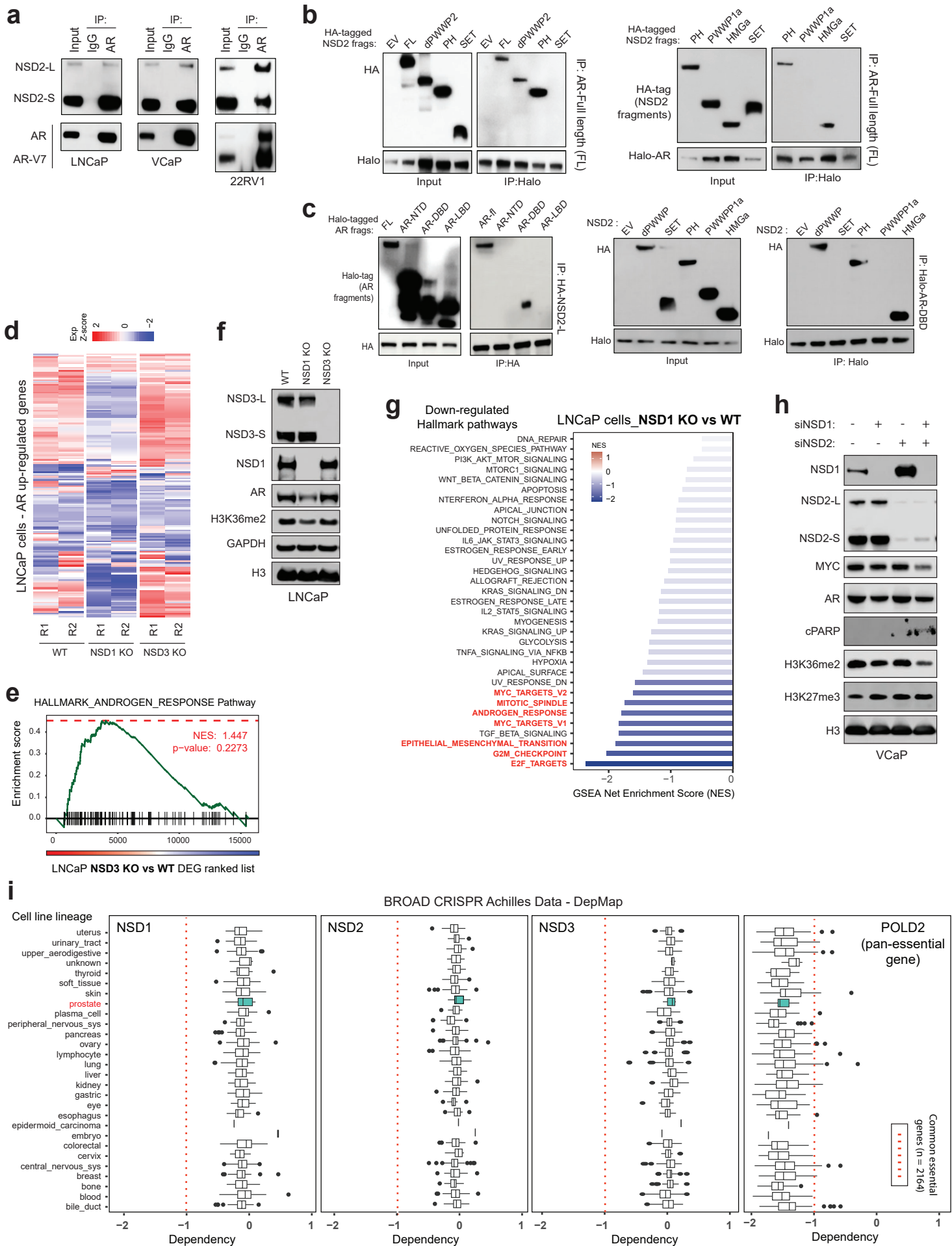

Figure S6

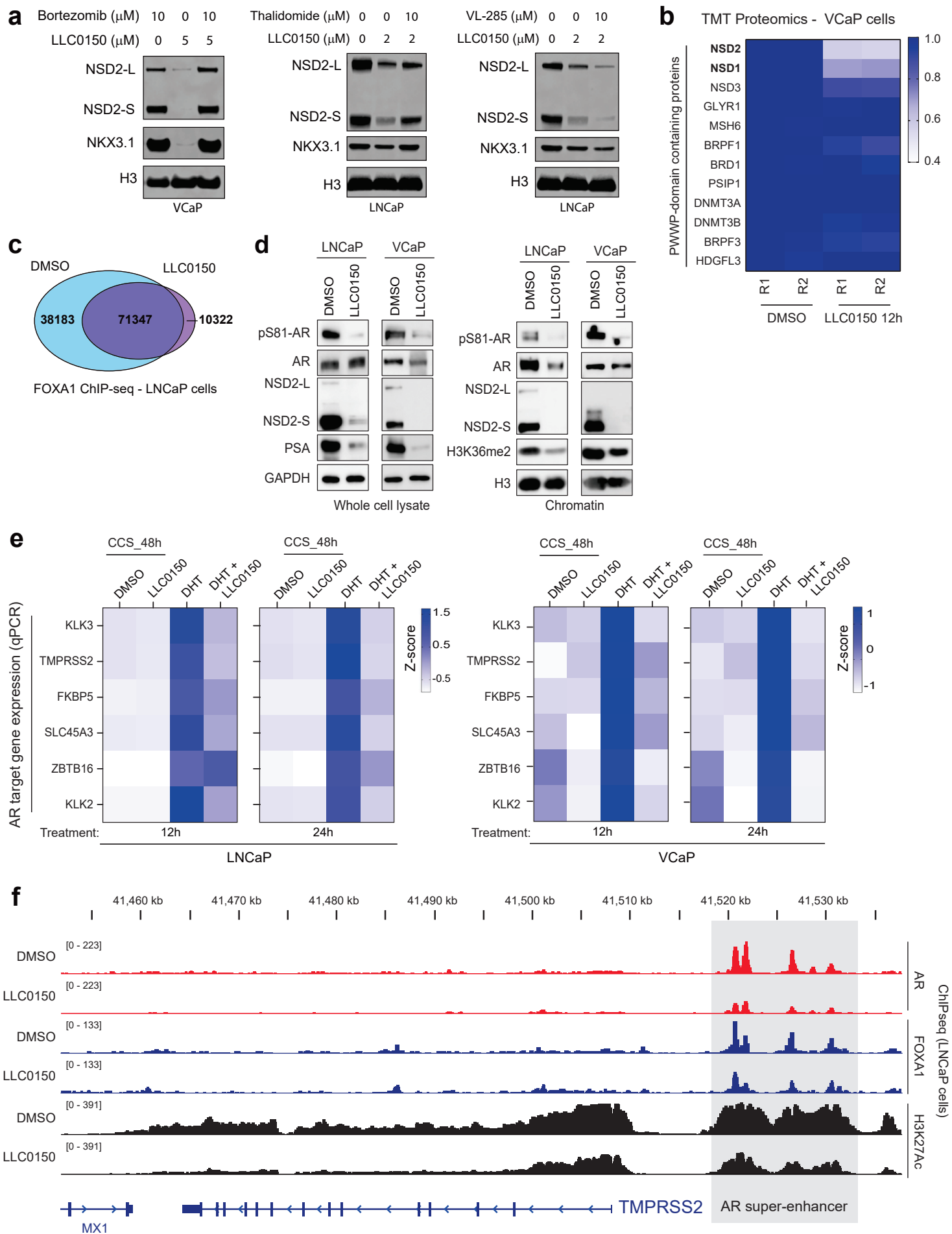

Figure S7

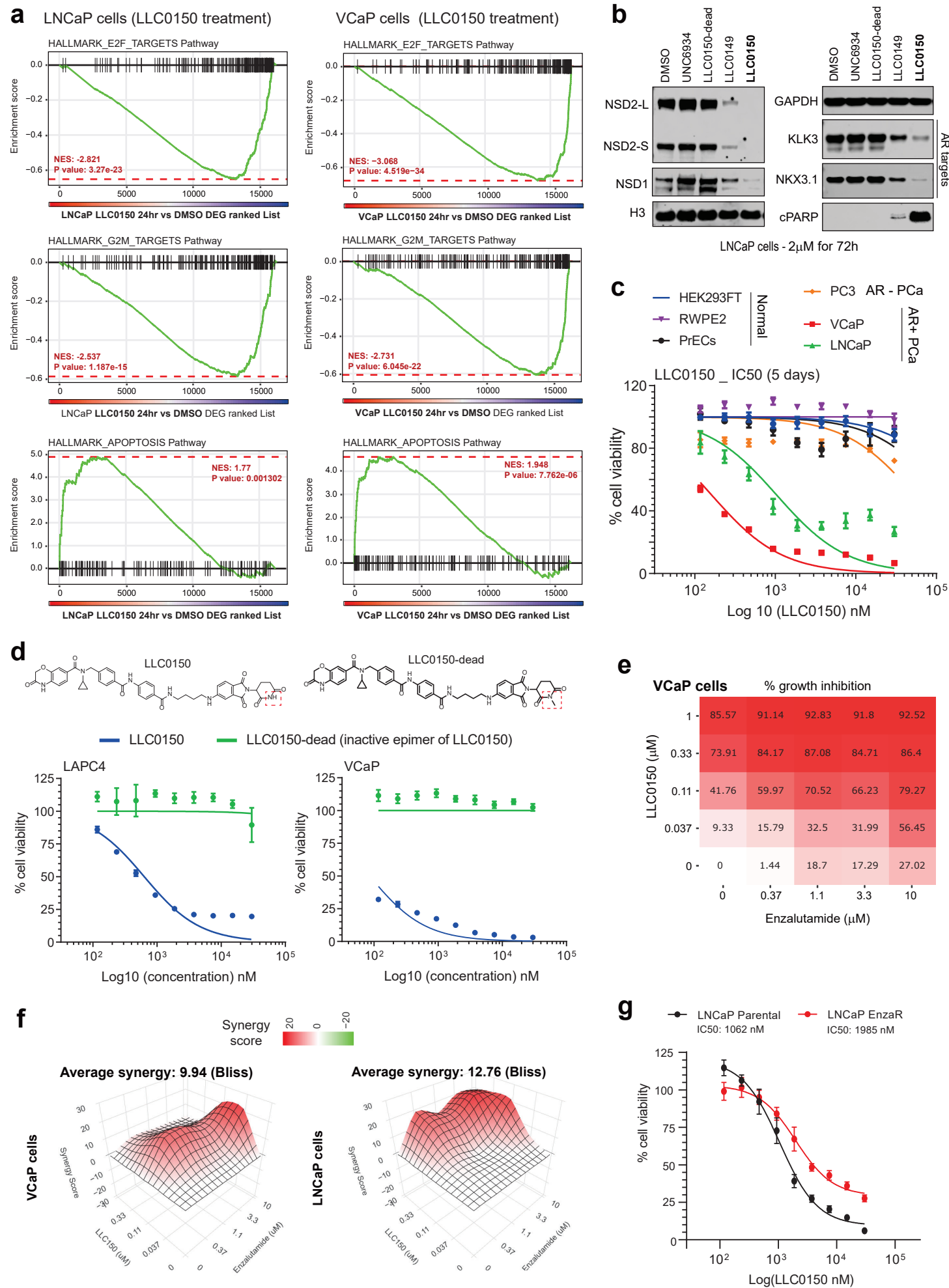

Supplement: Supplement 1 — Figure S1: Generation and characterization of the endogenous mCherry-PSA AR reporter cell lines. a) Schematic representation of the workflow of LNCaP-mCherry-PSA AR reporter cell line generation. b) DNA gel electrophoresis image showing the exogenously inserted mCherry amplicon in the LNCaP-mCherry-PSA lines. Clones 1 and 2 were used for the functional CRISPR screen. c) Sanger sequencing chromatograms of the PCR amplicon from reporter cells in panel (b) showing the KLK3/PSA gene promoter and exon 1 start codon junctions. d) Representative brightfield and mCherry immunofluorescence images of the LNCaP-mCherry-PSA clone 1 treated with (top) AR-targeting siRNA or ASO’s (siAR and ASO AR respectively) or enzalutamide (bottom left). Reporter cells were also serum starved for 48h and stimulated with DHT (10nM for 12h) to showcase gain in signal (bottom right). e) Immunoblots of noted proteins in LNCaP reporter cells as in panel (d). f) Expression (qPCR) of noted genes in reporter monoclones treated as in panel (d) to manipulated AR signaling. g) Immunoblots of noted proteins, including the exogenously introduced mCherry protein, in LNCaP reported cells treated with AR-targeting epigenetic drugs. Total H3 is used as a loading control. h) Next-generation sequencing-based abundance of sgRNAs in the epigenetic-focused library used in the CRISPR screen highlighting some of the known epigenetic regulators of AR. Figure S2: NSD2 transcript and protein expression in primary patient specimens. a) Immunoblot of labeled proteins in a collection of AR+ and AR- prostate cell lines. GAPDH and H3 are used as a loading control. b) Quantitative-PCR (qPCR) of KLK3 expression in LNCaP NSD2 WT and KO cells stimulated with R1881 for 12 and 24 hours. HPRT1 is used as a loading control. c) Immunoblot of labeled proteins in LNCaP NSD2 WT and KO cells stimulated with DHT for 30 hours. d) UMAP plots from patient-matched normal and primary prostate cancer single-cell RNA-seq data. e) NSD2 and PCA3 t [file media-1.pdf]
