# Supplementary material for: NSD2 is a requisite subunit of the AR/FOXA1 neo-enhanceosome in promoting prostate tumorigenesis
Source: bioRxiv. 2024 Mar 1:2024.02.22.581560. Preprint. [Version 2] doi: 10.1101/2024.02.22.581560 (PMC10925163; doi:10.1101/2024.02.22.581560)
Supplement: Supplementary file 1 [file NIHPP2024.02.22.581560v2-supplement-5.pdf]

bioRxiv preprint doi: https://doi.org/10.1101/2024.02.22.581560; this version posted March 1, 2024. The copyright holder for this preprint (which was not certified by peer review) is the author/funder, who has granted bioRxiv a license to display the preprint in perpetuity. It is made available under aCC-BY 4.0 International license.

Figure S1: Generation and characterization of the endogenous mCherry-PSA AR reporter cell lines.

a) Schematic representation of the workflow of LNCaP-mCherry-PSA AR reporter cell line generation.
b) DNA gel electrophoresis image showing the exogenously inserted mCherry amplicon in the LNCaP-mCherry-PSA lines. Clones 1 and 2 were used for the functional CRISPR screen.
c) Sanger sequencing chromatograms of the PCR amplicon from reporter cells in panel (b) showing the KLK3/PSA gene promoter and exon 1 start codon junctions.
d) Representative brightfield and mCherry immunofluorescence images of the LNCaP-mCherry-PSA clone 1 treated with (top) AR-targeting siRNA or ASO's (siAR and ASO AR respectively) or enzalutamide (bottom left). Reporter cells were also serum starved for 48h and stimulated with DHT (10nM for 12h) to showcase gain in signal (bottom right).
e) Immunoblots of noted proteins in LNCaP reporter cells as in panel (d).
f) Expression (qPCR) of noted genes in reporter monoclones treated as in panel (d) to manipulated AR signaling.
g) Immunoblots of noted proteins, including the exogenously introduced mCherry protein, in LNCaP reported cells treated with AR-targeting epigenetic drugs. Total H3 is used as a loading control.
h) Next-generation sequencing-based abundance of sgRNAs in the epigenetic-focused library used in the CRISPR screen highlighting some of the known epigenetic regulators of AR.

Figure S2: NSD2 transcript and protein expression in primary patient specimens.

a) Immunoblot of labeled proteins in a collection of AR+ and AR- prostate cell lines. GAPDH and H3 are used as a loading control.
b) Quantitative-PCR (qPCR) of KLK3 expression in LNCaP NSD2 WT and KO cells stimulated with R1881 for 12 and 24 hours. HPRT1 is used as a loading control.
c) Immunoblot of labeled proteins in LNCaP NSD2 WT and KO cells stimulated with DHT for 30 hours.
d) UMAP plots from patient-matched normal and primary prostate cancer single-cell RNA-seq data.
e) NSD2 and PCA3 transcript expression in patient-matched normal and primary prostate cancer luminal epithelial cells (pseudo-bulk analyses from single cell data; n=15, Wilcoxon test).
f) Representative immunohistochemistry (IHC) images of NSD2 in normal, primary prostate cancer, and metastatic castration-resistant prostate cancer patient specimens.
g) Boxplot showing RNA expression of labeled genes in primary prostate cancer specimens (TCGA cohort) stratified by the Gleason score. (Kruskal-Wallis test).

bioRxiv preprint doi: https://doi.org/10.1101/2024.02.22.581560; this version posted March 1, 2024. The copyright holder for this preprint (which was not certified by peer review) is the author/funder, who has granted bioRxiv a license to display the preprint in perpetuity. It is made available under aCC-BY 4.0 International license.

Figure S3: Motif characterization of the NSD2-enabled AR neo-cistrome in prostate cancer cells.

a) *Left*: Schematic representation of the half-motif enrichment analysis. *Right*: Motif enrichment plot of AR-half motifs with neighboring motifs of other transcription factors at NSD2-dependent and independent AR sites in LNCaP cells.
b) Venn diagram showing overlaps between AR ChIP-seq sites in LHSAR cells with LacZ (control), FOXA1, HOXB13, FOXA1+HOXB13 overexpression.
c) Venn diagram showing overlap of AR cistromes (ChIP-seq) in normal prostate, primary prostate cancer, and castration-resistant prostate cancer specimens. (Pomerantz et. al. 2015 & 2020)
d) Motif fold-change heatmap in normal, primary cancer, and castration-resistant prostate cancer specimens.
e) Fold change and significance of HOMER motifs enriched within mCRPC cancer-specific AR sites over normal tissue-specific AR elements (data from Pomerantz, 2015 and 2020).
f) Boxplot showing H3K27Ac ChIP-seq read density at sites containing the ARE or the FOXA1:AR motif in normal and tumor patient samples (normal prostate, n=7; primary prostate cancer, n=13; castration-resistant prostate cancer - CRPC, n=15). In box plots, the center line shows the median, box edges mark quartiles 1-3, and whiskers span quartiles 1-3 $\pm$ 1.5 * interquartile range.
g) ChIP-seq read density tracks of AR and H3K27Ac within the SLC45A3 and TMPRSS2 loci in NSD2 WT and NSD2 KO LNCaP cells. Super-enhancer clusters are highlighted in a grey box.

Figure S4: Molecular characterization of the NSD2-rescued prostate cancer cells.

a) *Left*: Immunoblots of noted proteins upon long-term treatment with control siRNA (siNC) or NSD2-targeting siRNA (siNSD2). *Right*: Immunoblot of NSD2 in LNCaP and 22RV1 cells treated with a control sgRNA (NSD2 WT) or sgRNA targeting NSD2 (NSD2 KO). GAPDH and total histone H3 are used as loading controls.
b) *Left:* Representative images of colonies of control or NSD2-null LNCaP cells. *Right*: ImageJ-based quantification of stained colonies shown in the images to the left (two-sided t-test).
c) *Top*: Tumor volumes of 22RV1 parental or NSD2-KO+HA-tagged NSD2-L cell line-derived xenograft in mice. *Bottom*: Immunoblot of noted proteins from the 22RV1 xenograft tumors from the growth experiment at endpoint.
d) *Top, left*: Representative images of Boyden chambers showing invaded cells stained with calcein AM dye in the LNCaP NSD2 WT and KO or NSD2-L rescued lines. *Right*: Quantified fluorescence signal from invaded cells (two-way ANOVA and Tukey's test)
   Bottom: Immunoblot of listed proteins in wildtype or NSD2 KO LNCaP cells with stable exogenous overexpression of NSD2-L and/or NSD2-S isoforms. eGFP is used as control.
e) Immunoblots showing expression of the KLK3 (aka PSA) protein in the eGFP or NSD2 overexpressing LNCaP cells.
f) Immunoblots of noted proteins in the NSD2 wildtype (WT) or NSD2-knocked out (KO) LNCaP cells that are rescued with exogenous WT or SET domain-deleted NSD2 mutants.
g) Immunoblots of noted proteins in LNCaP cells overexpressing the hyper-catalytic NSD2 SET domain E1099K mutant.
h) Immunoblots of noted proteins in the 22RV1 NSD2-FKBP12-F36V engineered cell lines upon dTAG-13 treatment (0.5uM for 6h).

bioRxiv preprint doi: https://doi.org/10.1101/2024.02.22.581560; this version posted March 1, 2024. The copyright holder for this preprint (which was not certified by peer review) is the author/funder, who has granted bioRxiv a license to display the preprint in perpetuity. It is made available under aCC-BY 4.0 International license.

i) *Left*: Box plot showing tumor weights of 22RV1+NSD2-FKBP12-F36V xenografts at endpoint (day 18) upon treatment with dTAGv-1. *Right*: Tumor images at the endpoint from the animal growth studies.
j) Heatmap showing expression (qRT-PCR) of AR target genes in the 22RV1 NSD2-KO + NSD2-FKBP12-F36V cell line after 12h or 24h of treatment with dTAG-13.
k) Immunoblots of noted proteins in size-exclusion chromatography fractions of nuclear lysate extracted from wild-type LNCaP cells. Fractions containing the AR protein are marked.

Figure S5: Fragment-based NSD2–AR co-immunoprecipitation and characterization of the NSD paralog knockout prostate cancer cells.

a) Immunoblots of indicated proteins upon co-immunoprecipitation of AR in prostate cancer cells.
b) Immunoblots of indicated proteins upon immunoprecipitation of exogenously expressed Halo-tagged full-length AR protein in HEK293FT cells that express HA-tagged NSD2 fragments. Both input (left) and immunoprecipitation (right) blots are shown.
c) *Left*: Immunoblots of HA-tag-based immunoprecipitation of full length NSD2 in HEK293FT cells that express the Halo-tagged AR protein fragments. *Right*: Immunoblots of Halo-tag-based immunoprecipitation of the DNA-bidning domain (DBD) of AR in HEK293FT cells that overexpress different HA-tagged NSD2 fragments. For both experiments, input and immunoprecipitation blots are shown.
d) Heatmap of AR upregulated genes (z-score) in NSD1 or NSD3 knockout (KO) LNCaP cells.
e) GSEA plots for AR-regulated genes using the fold change rank-ordered genes from LNCaP NSD3 knock-out (NSD3 KO) vs control cell lines. DEGS, differentially expressed genes.
f) Immunoblot of indicated proteins in NSD1 or NSD3-deficient LNCaP cells.
g) GSEA net enrichment score (NES) plot of downregulated hallmark pathways in LNCaP NSD1 knocked out (KO) vs wild-type control cells.
h) Immunoblot of indicated proteins upon treatment with NSD1 and NSD2-targeting siRNAs (labelled as siNSD1 and siNSD2) independently or in combination in VCaP cells.
i) Dependency map (DepMap) plots showing the dependency scores for NSD1, NSD2, NSD3, and POLD2 (positive control; pan-essential gene) across cell lines from distinct originating tissues. The red dotted line indicates pan-essentiality z-score cutoff.

Figure S6: Mechanistic characterization of the NSD1/2 PROTAC degrader LLC0150.

a) Immunoblots of indicated proteins in VCaP and LNCaP cells pre-treated with bortezomib, thalidomide, or VL-285 followed by treatment with LLC0150 at noted concentrations.
b) Heatmap of relative abundance of several PWWP-domain-containing proteins detected via Tandem Mass Tag (TMT) based quantitative MS upon 12h treatment with LLC0150 in VCaP cells.
c) Genome-wide changes in FOXA1 ChIP-seq peaks in LNCaP cells treated with LLC0150 (2uM for 48h).
d) Immunoblots of noted proteins in whole-cell or chromatin lysates from VCaP and LNCaP cells treated with LLC0150 (2uM) for 24h.
e) Heatmap of z-score normalized expression (qRT-PCR) of AR target genes in LNCaP and VCaP cells treated with LLC0150 followed by DHT stimulation (10nM for 24h). Treatment with DHT alone is used a control.

bioRxiv preprint doi: https://doi.org/10.1101/2024.02.22.581560; this version posted March 1, 2024. The copyright holder for this preprint (which was not certified by peer review) is the author/funder, who has granted bioRxiv a license to display the preprint in perpetuity. It is made available under aCC-BY 4.0 International license.

f) Read density ChIP-seq tracks of AR, FOXA1, and H3K27Ac within the TMPRSS2 super-enhancer in LNCaP cells treated with LLC0150 (2uM for 24h). Super-enhancer cluster is highlighted in a grey box.

Figure S7: Transcriptomic effect and drug synergism of LLC0150 in prostate cancer cells.

a) GSEA plots for E2F, G2M, and apoptosis pathway genes using the fold change rank-ordered genes from the LLC0150 vs DMSO treated LNCaP (left) or VCaP (right) cell lines. DEGS, differentially expressed genes.
b) Immunoblot of noted proteins in LNCaP cells treated with LLC0150 (2uM for 72h), dead-analog (LLC0150-dead), or the warhead alone (UNC6934). LLC0149 is an independent NSD1/2 PROTAC.
c) Dose-response curves of LLC0150 in normal prostate, AR-positive, or AR-negative prostate cancer cell lines at the indicated concentrations for five days.
d) Dose-response curves of LLC0150 and its inactive epimer control (LLC0150-dead) in LAPC4 and VCaP cell lines.
e) Percent growth inhibition (Cell-titer Glo) of VCaP cells upon co-treatment with varying concentrations of LLC0150 and enzalutamide for 5 days.
f) 3D synergy plots of LLC0150 and enzalutamide co-treated LNCaP and VCaP cells. Red peaks in the 3D plots denote synergy with the average synergy scores noted above.
g) Dose-response curves of LLC0150 in LNCaP parental and enzalutamide-resistant cell lines at varying concentrations for five days. Half maximal inhibitory concentration (IC50) at noted.

Table S1: List of known motifs enriched in the NSD2-dependent AR elements along with their HOMER statistics.

Table S2: List of known motifs enriched in the NSD2-independent AR elements along with their HOMER statistics.

Table S3: Antiproliferative half-maximal inhibitory concentration of LLC0150 across human-derived normal and cancer cell lines.
